# Are Biobased Plastics Green Alternatives?—A Critical Review

**DOI:** 10.3390/ijerph18157729

**Published:** 2021-07-21

**Authors:** Diogo A. Ferreira-Filipe, Ana Paço, Armando C. Duarte, Teresa Rocha-Santos, Ana L. Patrício Silva

**Affiliations:** 1 Department of Chemistry, University of Aveiro, 3810-193 Aveiro, Portugal; 2 Centre for Environmental and Marine Studies (CESAM) & Department of Chemistry, University of Aveiro, 3810-193 Aveiro, Portugal; anapaco@ua.pt (A.P.); aduarte@ua.pt (A.C.D.); ter.alex@ua.pt (T.R.-S.); 3 Centre for Environmental and Marine Studies (CESAM) & Department of Biology, University of Aveiro, 3810-193 Aveiro, Portugal; ana.luisa.silva@ua.pt

**Keywords:** plastic pollution, bioplastics, circular economy, biodegradation, sustainability

## Abstract

Environmental sustainability is driving an intense search for “green materials”. Biobased plastics have emerged as a promising alternative. Their building blocks can now be obtained from diverse biomass, by-products, and organic residues due to the advances in biorefineries and bioprocessing technologies, decreasing the demand for fossil fuel resources and carbon footprint. Novel biobased polymers with high added value and improved properties and functionalities have been developed to apply diverse economic sectors. However, the real opportunities and risks of such novel biobased plastic solutions have raised scientific and public awareness. This paper provides a critical review on the recent advances in biobased polymers chemistry and emerging (bio)technologies that underpin their production and discusses the potential for biodegradation, recycling, environmental safety, and toxicity of these biobased solutions.

## 1. Introduction

Since the introduction of plastics into the markets, their role in the world economy has grown immensely, now being omnipresent in several sectors, including construction, agriculture, medicine, and many others [1]. Diversity, malleability, durability, and a high degree of personalization are among plastics’ best qualities, leading the dependence upon these materials to naturally increase throughout the last century. This preference, together with the growth in population during this period, has led to massive production of these materials, resulting in equally huge waste generation and greenhouse gas emissions (GHG) [2,3]. In 2019, plastics production accounted for 10% of the global fossil feedstocks and reached a global production of approximately 370 million tons (Mt) [4,5]. A global generation of 150 Mt of post-consumer plastic waste and an emission of 390 Mt of CO_2_ were estimated in a World Economic Forum report for the year 2012 alone, and it should be noted that since then, plastic production has steadily increased [6]. If plastic usage continues at such a rate, plastics are expected to account for 20% of total fossil oil consumption and 15% of the total carbon budget, compared to approximately 1% at the time of writing that report. These numbers can be aggravated if we consider pandemic scenarios without implementing sustainable solutions [7].

Waste management infrastructures are still failing to cope with the waste generated from the continuous production and consumption of plastics, contributing to intensive loads of plastic waste ending up improperly managed [4]. Ideally, the plastics economy should be circularized to reduce plastic pollution worldwide; however, a significant share of plastic waste (around 79%) end up in landfills or improperly discarded in natural environments [1,8]. There, they can persist for hundreds to thousands of years, threatening animal and human health and affecting the balance of ecosystems [9,10].

To solve these shortcomings and reduce the plastic economy’s strain in the areas of environmental pollution and climate change, the modern plastics economy must be converted into a sustainable, circular framework [11]. Such a transition was prioritized by the United Nations in their 2030 Agenda for Sustainable Development, with goals such as 11 to 14 highlighting the need for the widespread implementation of measures to increase balance and sustainability in resource exploration and waste generation, and the importance of said measures for both environmental issues, such as ecosystem pollution and climate change, and societal issues, such as social cohesion and precarity, which can draw heavily from the former [12]. Several advances have been made, for example, in plastics recycling, with new technologies increasing the amount of plastic types that can be reconverted. Still, perhaps the most promising of these advances are biobased plastics [13,14]. However, focusing only on the fact that this next generation of “green” plastics can be produced free from fossil fuel intervention might be mistaking the forest for the trees, perhaps conveniently ignoring (in a purely market-oriented perspective) the issues of plastic recycling and reconversion, which are vital for the circularization of the plastics market, as well as those of environmental friendliness, to promote the marketable idea that these “green” polymers are the solution to humanity’s plastics woes [15].

This critical review is focused on the recent advances in biobased polymers chemistry and emerging (bio)technologies that underpin their production, addressing their opportunities and challenges when envisioning a sustainable and circular economy. It also discusses the potential biodegradation, environmental safety, and toxicity of these biobased solutions.

## 2. Plastic Pollution: A Social, Economic, and Environmental Problem

Since the 1950s, the volume of produced plastics has increased dramatically, from 2 Mt per year to 370 Mt in 2019, an over 190-fold increase that dwarfs the roughly tripling of the human population in the same timeframe [1,16]. Meanwhile, only 600 Mt of all the estimated virgin plastics produced ended up being recycled, with the vast majority being landfilled instead [1]. Plastic waste processing infrastructures worldwide have, therefore, proven incapable of adequately dealing with the sheer amount of incoming residual plastics, courtesy of today’s largely linear plastics economy, which emphasizes continuous production of new plastic over reconversion of used materials. On the other hand, even if the infrastructure in place could deal with the entirety of the incoming waste volume, the ability to recycle the plastics would be limited by the available methods of sorting and recyclability, which limit yields and, consequently, economic attractiveness. For instance, the different melting and glass transition temperatures of biobased PLA (Polylactic Acid) and fuel-based PET (Polyethylene Terephthalate) can interfere with drying and processing steps, resulting in lower-quality recycled PET [17,18]. Considering the economic point of view, it is also essential to keep in mind that failure to recycle plastics costs EUR 105 billion in the EU alone [19]. As such, this is a problem with multiple fronts beyond just the scientific, with economic, political, and social factors that must be dealt with to curb plastic pollution and the contamination of the ecosystems and food stocks, helping to minimize financial losses while at it.

In addition, it is vital to encompass other regions’ socioeconomic contexts to minimize plastic pollution and leakage. The EU is one of the richest areas of the world, but pales, population-wise, compared to the current developing regions, such as Brazil (over 212 million) and India (over 1380 million), put together [20]. In addition, whereas European citizens might be more economically comfortable and aware of plastics’ environmental footprint, developing regions are busy playing catch-up socioeconomically and thus less capable of implementing the sweeping reforms and infrastructure needed to deal with a tremendous waste output, especially when considering the lack of immediate economic benefits [21]. The Brazilian government’s position on Amazon development is a prime example of promoting economic opportunity near a vital ecosystem, with possible disastrous ecological consequences [22]. Rapid populational growth and a focus on exploration and economic development, combined with severe waste processing shortcomings, turn communities such as Manaus, population 2.2 million, in the Middle Amazon Basin, into waste generation behemoths; the result is a (conservatively) estimated 180,000 Mt of plastic wastes discarded into Amazonian environments yearly. Effects of this waste mismanagement might already be popping up downstream, with reports of fish, sea anemones, and stingrays being affected by plastic debris, the former in the Amazon River Estuary and the latter two from the Amazon Coast [23,24,25]. India also has quite the predicament, with estimates ranging between 4.8 and 12.7 Mt of discarded plastic entering the ocean yearly; this environmental situation is not helped by the fact India is crossed by heavily polluted rivers from other Asian countries, and that the Indian Ocean is also bordered by 10 of the 20 biggest plastic polluting nations worldwide [26]. Additionally, despite a growing interest in the long-lasting effects of environmental plastic pollution, the country’s waste management and regulation situation is expected to remain dire, thanks to high levels of single-use plastic consumption, ineffective legislation, insufficient infrastructure, and the low prioritization of this problem. Slowly, legislation is being enacted to reduce this problem, but great challenges remain for India in this regard.

**Figure 1:**
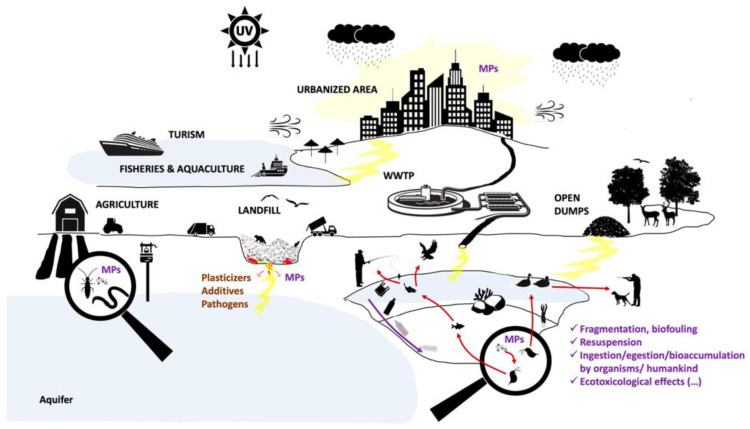
Schematic representation of sources, fate, and effects of plastic pollution on environmental and human health. MPs—Microplastics; UV- Ultra Violet (radiation); WWTP—Wastewater Treatment Plants.

Plastic pollution of the environment entails a wide range of negative consequences to animal and human health (Figure 1) [10]. For instance, due to their hydrophobic surface and longer half-life than most natural substrates, plastics in the environment slowly start being colonized by a diverse microbial community of heterotrophs, autotrophs, predators, pathogens, and symbionts, constituting the “Plastisphere” [27]. Such plastics and plastisphere can, therefore, promote the distribution of potentially non-native/allochthonous organisms/pathogens to other environments. In addition, plastic waste accumulation in soil systems can create a conducive environment for biological disease vectors [28] and affect water percolation and normal soils aeration, with repercussions on land productivity, as reviewed by Alabi et al. [29]. In addition, organisms can interact with plastic wastes: more than 260 different species of vertebrate and invertebrate animals were reported to have ingested plastics or have gotten entangled by plastic or plastic products, resulting in more than 400,000 deaths. Additionally, ingestion of plastic wastes/debris by animals often induces physiological effects such as perforation of digestive tracts, false satiation, and obstipation [30,31].

Regardless of initial dimensions, plastic debris can suffer degradation to various degrees in natural environments, slowly becoming smaller (from micro- to nanosized) and bioavailable to small-sized organisms [32]. This problem is amplified by the fact that plastics debris does not resist natural transport when in the environment, in other words meeting no borders. Plastic debris has been found in remote or guarded environments such as human-protected sanctuaries, such as the Pelagos Sanctuary in the Mediterranean Sea surrounding Corsica (France), or Gray’s Reef, off the coast of Georgia, USA, and Trindade, an island part of a remote Brazilian archipelago in the Atlantic, courtesy of economic and touristic activities for the former two, and the South Atlantic Gyre for the latter [33,34,35]. The ease of migration of this debris can pose an urgent threat to the health of watched and endangered species and, consequently, to the health of their ecosystems as a whole.

The effect of microplastics and nanoplastics (microplastics: 1 um–5 mm in size; nanoplastics: <1 um in size, with colloidal behavior [36]) on organisms and human health remain largely unknown; notwithstanding, studies conducted in controlled conditions on various organisms, including human and other animal cells, point to harmful effects when these are exposed to concentrations higher than reported in the field, thus exposing the potentially detrimental effect of these materials [37]. For example, both in vertebrates and invertebrates (with different feeding guilds), microplastics were found to affect feeding patterns, and therefore energy availability at best, or to trigger more severe symptoms in worse case scenarios—these can include severe inflammations and the triggering of stress pathways, endocrine disruptions, reduction in reproductive performance or even death events [22,38,39,40].

Given the tendency of persistence of these particles in organisms’ guts or other organs, bioaccumulation can also result in the effective poisoning of entire food webs, on which many human populations also rely. Humans are exposed to microplastics through various media, but their potential toxic effects still remain largely uncovered [41], although these materials seem to be able to trigger a range of inflammatory and cytotoxic events in human cells [42].

The risks plastic and microplastic ingestion pose for the ecosystem, and public health is even broader, however. Although plastic debris is considered biochemically inert, plastic additives are incorporated during manufacturing processes to improve plastics properties [43]. Furthermore, plastic debris can also act as a vector for other harmful chemical compounds such as heavy metals and biological pathogens, such as *Vibrio cholerae* and harmful algal bloom-generating organisms [44]. Plastic additives and/or absorbed contaminants can then leach out and eventually percolate into various environmental compartments, decreasing soil and water quality and inducing adverse chemical effects (summing up to the physical effects) on terrestrial and aquatic biota at different levels of biological organization [45].

Thus, the increase of plastic matter in ecosystems, the resulting incomplete and unsafe degradation into small-sized particles such as microplastics, their spread in the environments, and the resulting increased bioavailability to wide food webs become a severe health risk for chronically neglected ecosystems and public health.

## 3. Biobased Plastics and Circular Bioeconomy—The Road Ahead

Despite the various benefits plastics have in society, problems with plastic pollution (originating in waste or not) are some of the biggest challenges of our time. Once in the environment, plastic debris is somewhat difficult to recover. Research indicates that the best strategies for recovery consist in focusing on coastal areas, but in the EU alone, one of the regions in the world with the highest share of recycled plastic, those efforts can cost an estimated yearly EUR 630 million—a sum that will not turn a profit or reduce future economic damage, making it more challenging to approve and raise funding for these initiatives [19,46]. Throughout the last decades, plastics have become not only commonplace but entirely essential to a wide diversity of economic sectors, to the point that a carpet ban on these materials for the sake of the environment just is not feasible. Thus, one of the most valuable solutions to mitigate plastic litter inputs while restoring natural environments is by source-reduction and effective waste management to engage a more circular plastics economy. Beyond new waste processing methods, which are arguably not enough to sustain the ever-growing demand for these materials and the resulting influx of waste, the production of fossil fuel-independent plastics is also being touted as one of the key solutions in the plastics market reconversion that needs to occur in the coming years or decades [47].

Biobased plastics, as they have been dubbed, can be obtained from different renewable resources (e.g., plant-, algae-, residues-based) and, according to cradle-to-grave life cycle assessments, they seem to be generally advantageous in terms of saving fossil resources and reducing GHG emissions, as reviewed by Hatti-Kaul et al. [48]. As an example, significant savings of fossil fuel (40–50%) and GHG emissions (45–55%) have been reported for PEF (polyethylene furanoate) production when compared to PET (polyethylene terephthalate) [49]. Despite their apparent environmental attractiveness, biobased plastics currently account for merely 1% of the overall plastics market, or 3.8 Mt, although significant gains are expected in coming years [50]. These new materials must play catch-up against a well-established industry with over half a century of research, development and dominating market presence to its name—conventional petrochemical plastics have been continuously refined over the years to achieve the ideal properties for a range of different uses. Meanwhile, biobased plastics sometimes fall short when it comes to physical and chemical properties, highlighting the need for further research and funding and again hurting their short-term viability.

Notwithstanding, these new materials boast more attractive properties than the traditional alternatives. Still, considering the example of PEF, this polymer offers better performances reported for qualities such as permeability to oxygen and carbon dioxide than its fossil-based counterpart/competitor, PET [51]. Still, if a wider substitution of petrochemical plastics by biobased alternatives is to be achieved, biobased polymers with properties on par with other types of plastics must be developed. To that end, legislative and regulatory action is needed to boost the attractiveness of these emerging markets, thus incentivizing research and investment, which are often bottlenecks in biotechnological industries, especially in biobased instances such as this one [52].

Doing so will allow for better characterization and streamlining of production and end-of-life processes for these emerging biobased alternatives, such as those presented in Table 1, thus easing their entry into the broader markets.

Slowly but surely, governments are realizing the vital importance of the reconversion of the plastics economy away from fossil fuel exploration. In 2016, the French government published a decree on energy transition and green growth mandating the use of bioplastics in certain packaging applications, specifically biobased and home composting polymers [67]; the European Union, despite as of yet lacking specific legislation comprehensively regulating biobased, biodegradable, and compostable plastics, introduced in its European Green Deal and Circular Economy Action Plan (2019 and 2020, respectively) a policy framework regarding the main issues of sourcing, labelling and uses of these materials [68]; the United Kingdom, on the other hand, claims commitment to the tackling of plastic pollution, but still raises pertinent concerns with production and waste management, and highlights the need for more research to explore this issue further [69]. However, the legislation seems limited, and these deliberations seem to be only the exception to the rule [70]. Should these initiatives succeed and be adopted by more and more authorities, however, the biobased plastic sector can expect an increasingly favorable regulatory situation compared with traditional plastics’ going forward, adding the factor of economic attractiveness to the ecological perspective.

## 4. Biobased Plastics: Environmentally Friendly or Possible Foe?

**Figure 2:**
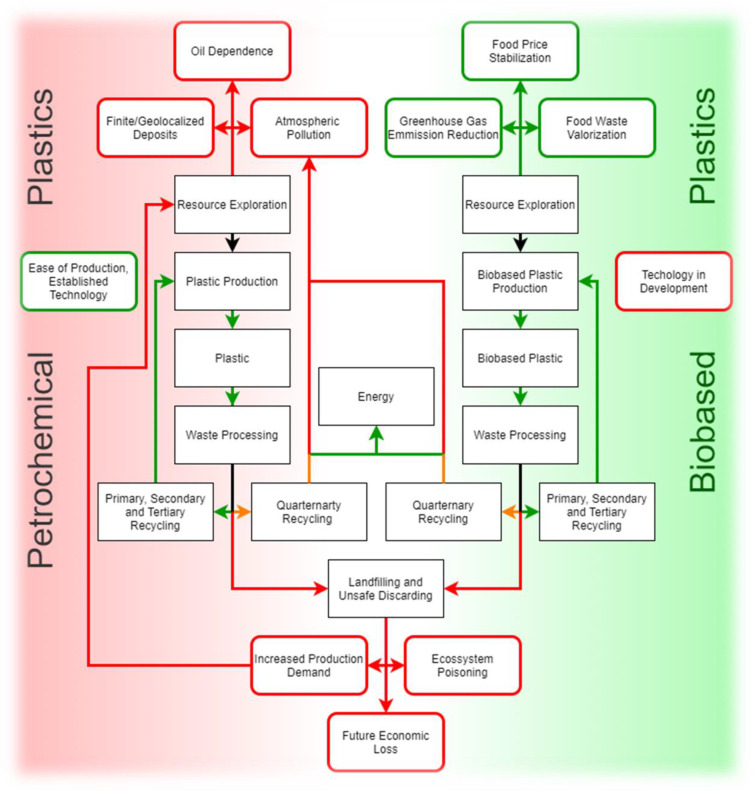
Schematic representation of petrochemical and biobased plastic life cycles, denoting some positive (green arrows) and negative (red arrows) effects of their use and disposal options.

Biobased plastics are touted as solutions to the environmental problems caused by conventional plastics production and waste (mis)management. However, they might come with a handful of environmental downsides of their own, and ignoring them can hinder the potential they have to curb the plastics economy’s large environmental footprint (Figure 2).

The environmental attractiveness of current biobased plastics remains controversial among academics and different stakeholders. Current production patterns for biobased plastics still presents considerable limitations that underline their weaknesses in the markets (Table 2). The production of biobased plastics remains associated with energy requirements (with most being dependent on fossil fuel resources), leading to controversies regarding their carbon emissions. For example, and as reviewed by Gerassimidou et al. [71], ethanol production from corn can be more energy-intensive than petrochemical plastic resin production, but the production of bio-PE leads to approximately 140% savings in CO2eq compared to high-density PE derived from fossil resources. In addition, woody feedstocks are highly lignocellulosic and resistant to degradation, so their conversion to a bio-based polymer resin requires an integrated biorefining process that involves the pre-treatment, enzymatic hydrolysis, fermentation, and further processing to iso-butanol (i.e., the starting monomer of bio-based plastic), which produces more GHG emissions and higher ecotoxicological impacts when compared with fossil-based plastics [72]. The replacement of fuel-based energy by renewable energy sources (e.g., solar, hydro, wind) and the development of microorganisms/enzymes to improve bioprocessing can reduce such limitations.

The use of chemical compounds (additives/fillers) at the polymerization stage of biobased polymer resins can impact environmental and human health. For example, acetyl tributyl citrate (ATBC) or polyethylene glycol (PEG) may be intentionally added to deal with PLA’s brittleness, high oxygen permeation, and poor thermal properties [73], which can aggravate both their biodegradability and ecotoxicity if discarded in open environments. Applying less toxic compounds, such as nanoclays and environmentally friendly nanocomposites (due to advances in nanotechnology as further discussed in Section 4), can improve biobased plastics properties [74].

End-of-life processing options for biobased plastics also raises environmental and economic concerns, as they are often misunderstood [15]. Although biobased plastics are, as the name indicates, plastics derived from renewable biological resources, that does not mean that their biodegradability is guaranteed. Some biobased plastics present resistance to degradation, such as PEF, some PLA options, Bio-PE, and Bio-PET, among others (Table 1). Hence, carelessly branding biobased plastics as green plastics might instill the wrong ideas in the minds of the consumers—the consequences of discarding these plastics, biodegradable or not, might be unintentionally ignored by the consumer lulled by the false sense of security given off by that green branding [75]; even certified biodegradable plastics are so only under specific conditions (e.g., in industrial composting facilities/bioreactors). For instance, PHA is biodegradable, but the extent of such biodegradability in aquatic environments was shown to depend on the inorganic water composition, water temperature, and polymeric chemical structure [76]. Some PLA options can also be biodegradable, but if discarded in marine environments, such polymers can take centuries to break down (weight loss of 2.5% was observed in a simulated marine environment over 600 days) [77]. Thus, careless discarding of these polymeric materials into the environment could have virtually the same effect as the “environmentally harmful” traditional petrochemical plastics, with toxicity assays demonstrating in vivo and in vitro toxicity [78,79]. For example, for PLA, Souza et al. found cytotoxic and genotoxic effects on the common onion (*Allium cepa*) [80], whereas Adhikari et al. detected inhibition of microbial activity caused by PLA films after 84 days of incubation in soil [81]. Huerta-Lwanga et al. [82] found that 1% PLA in composts resulted in significant mortality in earthworms (*Lumbricus terrestris*). The toxicity of PLA can be attributed to additives that are included in polymerization to improve mechanical properties. For example, substances such as tributyl citrate or PEG are commonly added to PLA for plasticization; additionally, to improve impact resistance, isocyanates can also be added as chain extension agents by forming a polyurethane bond with the terminal hydroxyl group of PLA [83,84].

On large scales, these attitudes could end up offsetting any positive impact biobased plastics might achieve. To solve this problem, the consumer base must be thoroughly educated on these materials and their waste management practices. This might seem at first like an obvious point. However, its importance is backed up by data that suggests that consumers are somewhat unfamiliar with the concept of biobased plastics, which is in their minds is more associated with environmental issues rather than technical ones—keywords such as “biodegradable” and “environmentally friendly” being more linked to these plastics than “independent from oil”, one of their actual defining features, highlighting how easy it is to misrepresent biobased plastics [85].

Regulating authorities also have the responsibility to demand clear labelling to easily relay the proper disposal methods to the consumers to convert them into active participating members of the plastic waste processing infrastructure. For such correct labelling, international guidelines must also be updated. International standards specify the requirements for biodegradable plastics in composting, home composting, and soil or water compartments (e.g., EN 13432, ASTM D6400, Vinçotte OK Biodegradable Soil/Water). Typically, full biodegradation is assessed as the first tier of testing, and ecotoxicity is addressed as the second tier of testing [86]. However, as reviewed by Kjeldsen et al. [86], such international guidelines have several issues that can limit their reliability when attempting to predict biodegradation in environmental scenarios, such as limited methodology (primarily based on respirometry measurements), unrealistic testing conditions (e.g., aqueous/soil medium, controlled conditions or anaerobic digesting sludge), lack of guidance for employing different test materials (e.g., powder, film), insufficient statistical power from limited replicates (often <3), unsuitable procedures for aquatic environments, many related to wastewater treatment plants (WWTP) situation, and flaws in toxicity testing that are often based on single-(model) species assays, without considering the impact of plastic litter and potential persistent compounds from the biodegradation process on multispecies communities, biochemical processes and ecosystem functioning.

For example, Mater-Bi^®^ (a starch-based plastic) can achieve up to 80% biodegradation in 90 days in aerobic compost conditions (according to EN14045, ISO14851) [87,88]; however, in soil and aquatic conditions, this bio-based plastic only achieves 3.4% and 1.5% biodegradability, respectively, in the same timeframe [89]. Based on single-species tests, such bio-based plastics seem to present no ecotoxicity [90], though the effects at lower (cellular and biochemical level) and higher (community and ecosystem level) biological organization remain poorly covered.

In addition to the implementation of adequate guidelines and correct labelling, an adaptation of the existing recycling infrastructure is needed to accommodate these new materials, including new recycling procedures and sorting mechanisms, which can prove to be somewhat of a challenge given the (intentionally) similar characteristics between specific plastics and some candidates for substitutes [18]. For example, PLA can be applied in transparent bottles (visually like PET bottles) and can end up on PET recycling streams, and even a 2% contamination would interfere with drying and processing steps, resulting in poor-quality recycled PET (rPET).

This fact, in turn, puts pressure on the waste management facilities, and without support, they may be less than willing to accept these wastes. The United Kingdom’s government has recognized that facilities related to composting and anaerobic digestion sometimes show reluctance in even accepting the waste materials in the first place [69]. As such, adequate incentives are needed to update and expand the underlying recycling infrastructure to accommodate biobased plastics without risking causing possibly severe plastic pollution increases due to these new, conditionally, eco-friendly materials.

## 5. New Sources and (Bio)Technological Approaches for Improving Biobased Polymers Engineering and Properties

Recent advances in biorefinery and polymer chemistry have been applied to produce biobased solutions from alternative biomass (e.g., algae) and residues, with improved design, properties, and functionalities for their successful introduction to the markets and ensure their recyclability (chemical or mechanical).

The use of alternative biomass, by-products and wastes for green valorization provides a substantial ecological advantage when comparing with plant-based biomass sources, as they reduce arable land pressure and help lessen the issue of competition for food production, as well as the intensive use of fertilizers, pesticides and water, while reducing carbon footprints related to waste generation through their reuse as new raw materials [91]. Several compounds, such as lipids, flavonoids, lignocellulose, and phenolic compounds, can be extracted from agro-industrial, forest and even food wastes to produce high value-added biobased products via bioprocessing within a biorefinery framework (as reviewed by Patrício Silva, 2021 [17]). Concomitantly, chitin and chitosan have come up as value-added products that can be retrieved from industrial seafood waste, presenting several appreciated properties such as antimicrobial activity, chelation properties, film formation, and decent mechanical strength as a potential competitor for food packaging material [66,92,93,94]. Furthermore, it has also been used in edible coatings to enhance the shelf life of fresh produce or processed fruit, vegetables, poultry, and dairy products on a lab-scale without interfering with their sensory attributes.

In addition, algae-based biomass has been highlighted as an alternative approach to achieving sustainable plastic production while contributing to reduce the environmental footprint of production [95]. Algae (micro to macro) possess rapid growth, plasticity, reduced cultivation costs, and autotrophy that contribute to reducing the GHG emission by sequestering CO_2_ (up to 1.8 lb) and releasing oxygen (>75%) [96]). Polyhydroxyalkanoates (PHAs) and homopolymers, such as polyhydroxybutyrates (PHBs), can be algal-based, and both can present similar physicochemical and mechanical properties as their closest petrochemical counterparts (e.g., polypropylene, polyethylene terephthalate, and polyethylene) with potential applications in industry, agriculture, and packaging [97]. Microalgae can be used as biofillers to improve mechanical properties in novel thermoplastic biocompounds from gluten [98].

The production of polymer building blocks from biomass or residue components relies on enzymatic tools (as reviewed by Hatti-Kaul [48]), and enormous efforts have gone into the screening and development of enzymes that hydrolyze different components of the biomass/residues. This process is costly, however, mainly due to the high energy demand and biological activity. Consolidated bioprocessing that involves the development of microbial strains with engineered degrading activities or the development of co-cultures that would allow the direct conversion of the biomass/residue to the target molecule have been gaining momentum to overcome such limitations. Metabolic engineering strategies could also include pathway prioritizations by changing substrate preferences, managing redox balances, easing the transport of metabolites, and improving resistance to inhibitory factors such as reaction product concentration or pH, among others to achieve high product yields and selectivity [48].

Meanwhile, advances in nanotechnology have revealed its potential to play an essential role in the polymerization process to improve biobased plastics’ functionalities and properties. The inclusion of nanocomposites (e.g., nanoclays) in the polymerization process can result in materials with an improved balance between permeabilities for oxygen, carbon dioxide, nitrogen, and water vapor, with lower costs compared to other nanomaterials and chemical additives [74]. A significant contribution of such an application was observed with PLA microlayer films, which solved problems associated with loss of transparency and heat resistance by obtaining the flexibility required for packaging applications. Still, their environmental friendliness remains to be seen.

High-performance biobased polymers with desirable material features for recycling are also in demand. The glass transition temperature (Tg) is one of the most important thermal properties of amorphous plastic materials, determining their physical, mechanical, and rheological properties and, hence, their range of applications (as reviewed by [48]). Commercial biodegradable polymers generally have a low Tg—notably, for PHA, this parameter can reach a relatively low value when comparing to other plastics of −28 to −55 °C. [99,100]. However, the introduction of aromatic units (e.g., phenyl, phenoxy, and benzoyl) in the PHA chain can significantly increase the Tg of the polymer, which in some cases can then reach beyond the 20 °C threshold, depending on the aromatic group. Thus, improvements in Tg of aliphatic polyesters can be an effective strategy for increasing their performance and recycling possibilities and even their optical transparency. PEF presents yet another example—resorting to the use of the FDCA dimer 2,2′-bifuran-5,5′-dicarboxylate as the monomer can significantly improve the Tg of fully biobased PEF (from 86 to 107 °C), even though this parameter was already higher in this plastic than its competitor, PET (74 °C) [8].

Biobased polymers are getting closer to the reality of replacing their petrochemical counterparts than ever before, paving the way towards a more sustainable and circular economy. It is expected that soon, these materials will be used in several areas, from commodities to advanced applications, thanks to developments in biotechnology and bioprocessing.

## 6. Final Considerations

Despite as of this point facing a somewhat uphill battle to secure a significant foothold in the plastics market, in coming times, the biobased plastics sector is expected to grow, lifted by an increasingly environmentally aware consumer base and more forgiving regulatory circumstances, potentially helping reduce the carbon footprint associated with the whole plastics industry.

However, simply taking these new “green” plastics at face value is risking taking a step backwards in the fight against plastic-related pollution. Data indicate that consumers, even in regions thought to be highly developed and educated, seem to easily mischaracterize what terms relate to these new generation plastics. Before biobased plastics become truly commonplace, though, rules and regulations should be put in place that incentivize manufacturers to integrate environmental performance in the development of new polymers and demand rigorous toxicity and life-cycle safety assessments for the new products. Furthermore, when it comes to waste management, the regulatory frameworks must strongly enforce the reutilization and recycling routes for these new materials, or, as a last resource, the quaternary recycling to produce energy. This, in turn, means more investment will be necessary to properly integrate biobased plastic recycling methodology with the current capacities, given that sorting different plastics that were designed to behave similarly to existing ones is a hurdle that must be overcome to maximize the efficiency of the incoming plastics’ reconversion.

Landfilling is a waste management solution that must be avoided at all costs, and aggressive action against littering is a must, given that even the subset of plastics deemed biodegradable by the admittedly lacking regulations on this matter require somewhat specific environmental conditions to degrade in the environment safely; as such, biodegradable options might further risk lulling the consumers into a false sense of security concerning their ecological safety. Once again, the importance of education efforts is highlighted but shows that prioritizing biodegradability rather than biological production might be misguided. Some certified biodegradable materials are chemically harmful after said degradation. As such, since biodegradability only ever so slightly reduces the environmental harm of littered plastics, all the while limiting the circularization of the plastics economy by diminishing consumers’ worries about plastic discarding and landfilling rather than recycling, biobased plastic production might just be the better bet of the two to realize the ideal of a sustainable, circular plastics economy.

In sum, to truly begin to fix the problem of plastic pollution and its ramifications on the climate, ecosystems, and public health, the plastics economy must be rethought from a sustainable, circular, and low carbon perspective. Biobased plastics can emerge as tools with high potential for this conversion, although not without issues, both inherent and related to their relative novelty. As such, this subset of the plastics industry must be scaled up responsibly, always considering the economic, legislative, and social sides of this equation so that it may have the opportunity of truly fulfilling its perceived potential.

## Figures and Tables

**Table 1:** Production, usage, and end-of-life options for commercially available (or soon to be available) biobased polymers.

Polymer	Synthesis	Market Application	End-of-Life/Biodegradability	References
Agrobiofilm^®^	Formulated using a starch base complemented with renewable raw materials from vegetable oils.	Used as additives in horticultural and perennial soils	Biodegradable and intended for in situ degradation	[53]
Bio-PA11	Synthesized using 11-aminoundecanoic acid from castor oil	Automotive and fuel tubings, electrical components, coatings	Non-Biodegradable, Chemical Recycling and Mechanical Recycling	[54,55,56]
Bio-PE	Dehydration of bioethanol from glucose	Food Packaging, Automotive applications, toy production, cosmetics and other industrial and agricultural applications	Non-biodegradable, mechanical recycling	[56,57]
Bio-PEF	Derived from 2,5-furandicarboxylic acid, which can be generated entirely from sugars such as cellulose	Being developed as a competitor to PET, mostly for packaging applications	Non-biodegradable, enzymatic depolymerization	[51,56,58]
Bio-PET	Synthesized using bio-ethylene glycol or bio-terephthalic acid from Glucose and Fructose	Fibres and a variety of packaging applications	Non-biodegradable, chemical recycling, mechanical recycling and enzymatic depolymerization	[56,57,59]
Bio-PP	Butylene dehydration of bio-isobutanol from glucose	Not yet industrially produced, confidential pilot plant phase	Non-biodegradable, mechanical recycling	[56,57]
PBS	Produced with succinic acid derived from biomass	A variety of packaging applications, including food packaging, as well as agricultural mulch films	Biodegradable, chemical recycling and enzymatic depolymerization	[56,60,61]
PHA and PHB	Bioproduction within micro-algae, bacteria and archaea	Various packaging, agricultural and medical applications	Biodegradable, home and industrial composting, anaerobic digestion and chemical recycling	[56,62,63]
PLA	Derived from microbial-produced lactic acid	Food packaging, electronic components and 3D printing materials	Biodegradable, mechanical recycling, chemical recycling and industrial composting	[56,64,65]
Chitosan	Derived from exoskeletons of crustaceans, insects, cell walls of fungi and yeast.	Various packaging, agricultural and medical applications	Biodegradable, anaerobic digestion and chemical recycling	[66]

Bio-PA11—Bio-Polyamide; 1Bio-PE—Bio-Polyethylene; Bio-PEF—Bio-Polyethylene Furanoate; PET—Polyethylene Terephthalate; Bio-PET—Bio-Polyethylene Terephthalate; Bio-PP—Bio-Propylene; PBS—Polybutylene Succinate; PHA/B—Polyhydroxyalkanoate/Polyhydroxybutirate; PLA—Polylactic Acid.

**Table 2:** Summary of pros, cons, and emerging solutions regarding biobased plastics (from cradle to crate).

Pros	Cons	Emerging Solutions
▪ (Partly) based on natural feedstock ▪ Generally, lower GHG emissions ▪ Lower dependence on crude oil ▪ Favorable policy landscape (e.g., EU plastic strategy) ▪ Biodegradable options can simplify waste management and returns carbon to the soil, potentially mitigating plastic pollution	▪ Costly manufacturing ▪ (Partly) use of genetically modified organisms ▪ Use of arable land, fertilizers, and pesticides for crops (which results in soil erosion and degradation) ▪ Potential food competition ▪ Narrow processing window (e.g., lower melting temperature) ▪ Brittleness ▪ Thermal degradation ▪ Bioconversion requires a high amount of energy ▪ Potential for harmful effects on biota (similar to petrochemical counterparts) ▪ Potential to contaminate recycling streams ▪ Uncertainty regarding biodegradability in open environments (due to current and limited international guidelines for product certification)	▪ Biorefinery technology ▪ New strains of microorganisms/enzymes required to improve bioprocessing ▪ Algae, waste residues, by-products as sources to retrieve building blocks ▪ Implementation of renewable energy sources (e.g., solar, geothermic) for plastic production ▪ Advances in nanotechnology (e.g., application of nanocomposites such as clays) to improve physicochemical and mechanical properties ▪ Production of biobased plastics of pure polymers, or blended with compounds free of (eco)toxic effects ▪ Dedicated recycling streams and adequate labelling ▪ Appropriate and coordinated international guidelines for product certification ▪ Increased public awareness and education efforts ▪ Increase in financial programs for sustainable plastics production and management of wastes

GHG—Greenhouse Gas.
